# iDMET: network-based approach for integrating differential analysis of cancer metabolomics

**DOI:** 10.1186/s12859-022-05068-0

**Published:** 2022-11-28

**Authors:** Rira Matsuta, Hiroyuki Yamamoto, Masaru Tomita, Rintaro Saito

**Affiliations:** 1 Institute for Advanced Biosciences, Keio University, Tsuruoka, Yamagata 997-0052 Japan; 2 Systems Biology Program, Graduate School of Media and Governance, Keio University, Fujisawa, Kanagawa 252-8520 Japan; 3 Human Metabolome Technologies, Inc., 246-2 Mizukami, Kakuganji, Tsuruoka, Yamagata 997-0052 Japan

**Keywords:** Metabolomics, Data integration, Multi-laboratory comparison, Reproducibility, Odds ratio

## Abstract

**Background:**

Comprehensive metabolomic analyses have been conducted in various institutes and a large amount of metabolomic data are now publicly available. To help fully exploit such data and facilitate their interpretation, metabolomic data obtained from different facilities and different samples should be integrated and compared. However, large-scale integration of such data for biological discovery is challenging given that they are obtained from various types of sample at different facilities and by different measurement techniques, and the target metabolites and sensitivities to detect them also differ from study to study.

**Results:**

We developed iDMET, a network-based approach to integrate metabolomic data from different studies based on the differential metabolomic profiles between two groups, instead of the metabolite profiles themselves. As an application, we collected cancer metabolomic data from 27 previously published studies and integrated them using iDMET. A pair of metabolomic changes observed in the same disease from two studies were successfully connected in the network, and a new association between two drugs that may have similar effects on the metabolic reactions was discovered.

**Conclusions:**

We believe that iDMET is an efficient tool for integrating heterogeneous metabolomic data and discovering novel relationships between biological phenomena.

**Supplementary Information:**

The online version contains supplementary material available at 10.1186/s12859-022-05068-0.

## Background

In metabolomics, the comprehensive analysis of metabolites, multiple separation methods such as capillary electrophoresis (CE), liquid chromatography (LC), and gas chromatography (GC) have been developed. They are often used with one of the various types of mass spectrometer, such as time-of-flight (TOF), orbitrap, and triple-quadrupole (QqQ) mass spectrometers, which have different sensitivities [1]. Since the field of metabolomics was established, metabolomic data have been acquired by these analytical platforms for many research fields, such as biomarker discovery using human biological fluids and elucidation of drug mechanisms using animal models [2, 3]. In the future, it may be commonplace to integrate multiple metabolomic datasets and make biological inferences that could not be made from individual datasets. In fact, in the field of transcriptomics, databases and data analysis methods have been proposed, such as COXPRESdb to search for co-expressed genes from large datasets, and CellMontage to search for sample similarity from gene expression profiles [4, 5]. Although research on the integration of multiple metabolomic profiles from different studies has recently been initiated in the context of meta-analysis [6, 7], data analysis methods for integrating metabolomic data in general acquired by different analytical platforms have not been well studied. This is due to two main problems in metabolomic data, with the first one being rather common in other omics data as well.

The first problem is the reproducibility of the metabolite level measured at different facilities [8]. In mass spectrometry-based metabolomics, the peak area of each metabolite has often been used as its quantity for statistical analysis. The peak areas of metabolites from different studies cannot be simply gathered for subsequent statistical analyses because peak area depends not only on metabolite concentration but also on the sensitivity of mass spectrometry, which varies with different instruments. Many other factors including capillary replacement and elapsed time after start of measurement also have effects on the sensitivity [9]. To integrate metabolomic data acquired from different analytical conditions, normalization of the quantitative value of each metabolite using the corresponding stable isotope as an internal standard is essential. However, stable isotope reagents are very expensive, and it is practically difficult to prepare specific isotopes for each of the metabolites [10]. Recently, a data integration approach by using pooled QC samples with normalization has been applied in large-scale metabolomic studies [9, 11–13]. However, the application of this approach is limited to large-scale studies of human biofluid samples on the same instrument, such as cohort studies, and it is not applicable to the integration of metabolomic data acquired from different analytical conditions.

The second problem is the overlap of the sets of metabolites measured in different laboratories. In metabolomics, there are multiple separation methods such as CE, LC, and GC, so the targeted metabolites differ depending on which is selected [14]. Even if the separation analyses are the same, the sets of detected metabolites do not always match because the number of detected metabolites depends on the sensitivity of the mass spectrometer. For example, Bing et al. reported that only 126 metabolites were detected by at least two platforms among 1421 metabolites measured by Metabolon, Broad Institute, and Nightingale Health, and only 14 metabolites were detected on all three platforms [15]. This means that, in most cases, only a small proportion of metabolites remain after merging metabolomic data, which limits the number of situations in which we can use a merged metabolomic dataset.

To integrate multiple sets of metabolomic data while avoiding these two problems, we developed iDMET, a network-based approach to integrate metabolomic data from different studies. For the first problem of reproducibility, we referred to the paper by Izumi et al. They measured target and control samples at different laboratories and reported that the ratios of target sample to control sample were highly reproducible for many metabolites [16]. Therefore, we integrated different studies based on the differential metabolomic profiles between two groups, instead of the raw peak area, an approach similar to the one used in the “amanida” software package for meta-analysis [7]. We further avoided the second problem of low overlap of the metabolites among multiple studies by performing a pairwise approach that integrates one pair of two differential metabolomic profiles at a time, instead of integrating them all at once. iDMET is designed to conduct “data-driven” biology, which can be widely used to generate new hypotheses from various metabolomic data, as opposed to meta-analysis where collected metabolomic studies should focus on the same disease (or biological phenomenon) according to the specified hypothesis [6, 7]. Thus, iDMET may discover unexpected findings such as connections between different cancer types from the metabolomic data obtained at different facilities and from different samples.

As an actual application to cancer metabolomics, we collected metabolomic data from 27 articles or repositories published so far, and integrated them in iDMET. We focused on strong relationships between different studies and found results that led to novel biological inferences.

## Methods

### Literature collection

**Fig. 1:**
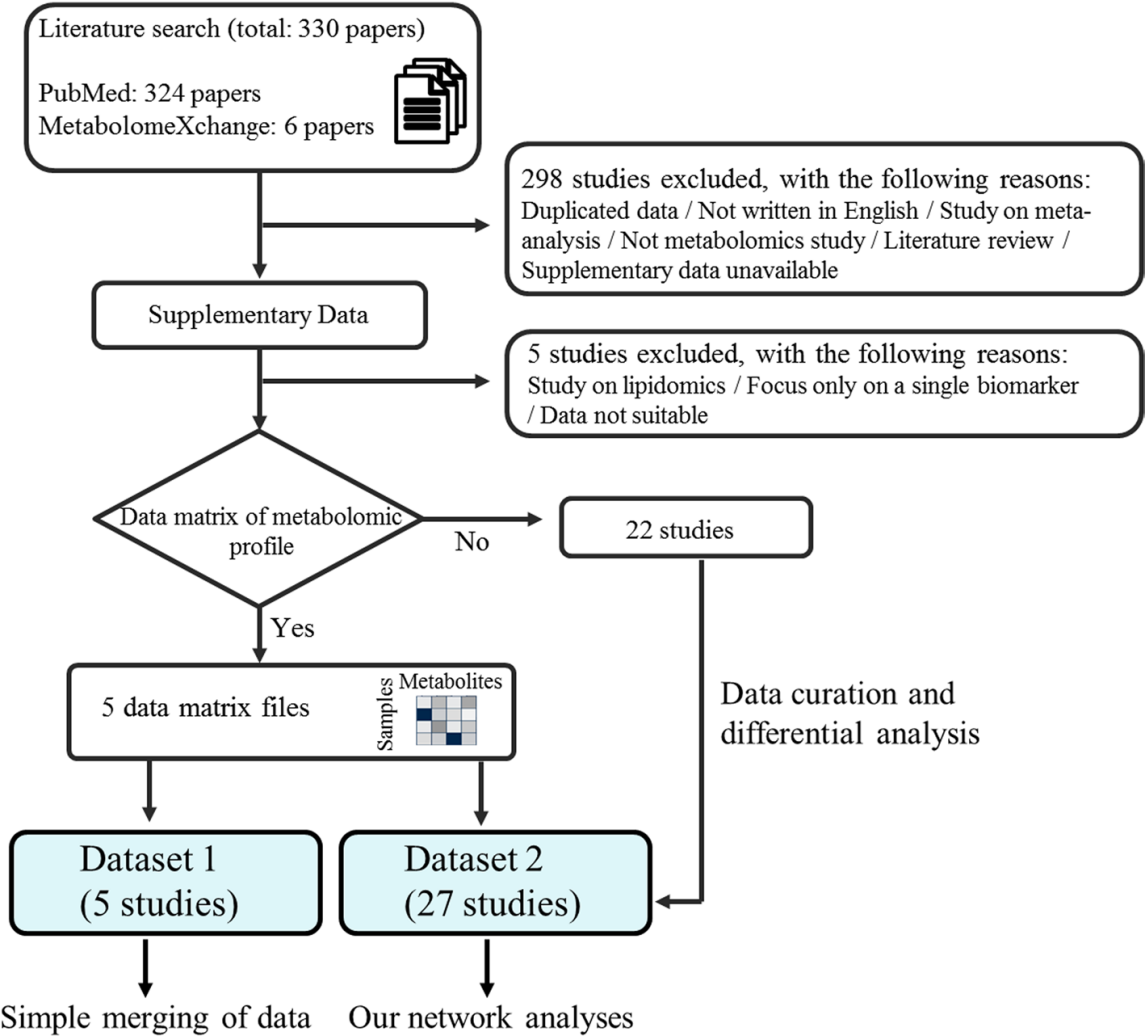
Workflow diagram for the collection and integration of metabolomic data from public resources. The literature search resulted in 324 hits and an additional six references were identified from the metabolome repository (MetabolomeXchange). Among the 330 studies identified by the initial search, we excluded 303 studies for the reasons shown in the figure. We finally selected 27 studies suitable for analysis. Among them, data matrices of metabolomic profiles were available for five studies, which were used to investigate whether simple merging of multiple sets of metabolomic data is efficient (left blue box at the bottom). Metabolomic data from all 27 studies were used for our newly proposed method (right blue box at the bottom). The details of the flow chart and how each of the datasets was used are described in the methods

A large number of studies related to cancer metabolomics have been conducted on multiple platforms. We searched for relevant entries in PubMed and MetabolomeXchange, an online portal of metabolomics repositories, which includes data from four different repositories, namely, MetaboLights [17], Metabolomics Workbench [18], Metabolomic Repository Bordeaux [19], and Metabonote [20]. The search terms included “Metabolomics,” “Metabolome”, “Tumor cells, Cultured,” and “Hypoxia” and their variants, and they were used initially to search for relevant literature in PubMed. In addition, “hypoxia, cancer” was used as a search term in the repository MetabolomeXchange. The entries found in PubMed (324 papers) and repositories (6 papers) using the above terms included those that are irrelevant or unsuitable for this study (Fig. 1). For instance, some studies focused on the structural biology of a metabolite, while others had a bioinformatics focus. Each article was manually curated to obtain and organize relevant information and to determine whether each article was suitable for our goal of integrated analysis.

The following criteria were used to select metabolomics datasets for this study: (1) metabolite data were available in a form amenable to reading or parsing computationally (text file or a common format of spreadsheet etc.), and (2) data were representative of the primary metabolomics technologies (e.g., nuclear magnetic resonance, gas chromatography mass spectrometry, liquid chromatography mass spectrometry). Quality control of metabolomic data was beyond the scope of our current work, so the quality of the metabolomic data depended on the quality control conducted in each study. Searches were performed in February 2020.

### Datasets

We finally selected 27 studies suitable for analysis (Fig. 1). Among them, data matrices of metabolomic profiles, where rows and columns of each matrix represent samples and metabolites, respectively, were available for five studies (Dataset 1) and were used to test the efficiency of simple merging and sample normalization of metabolomic profiles from multiple studies, where a metabolomic profile is a set of metabolite levels detected in a specific condition. For creating the data matrices, we used metabolite levels pre-processed by the original authors. Detailed descriptions of the data pre-processing methods (normalization, removing metabolites with large coefficient of variations, imputing missing values, etc.) can be found in Additional file 2: Table S2 as well as in the original articles. The simple merging is defined as vertical concatenation of data matrices of metabolomic profiles. Metabolomic data from all 27 studies (Dataset 2) were used for testing and evaluation of the iDMET method. A brief summary of the datasets is shown in Table 1. The computational framework for this study required data to be converted to a standardized Excel file format, where each column represents a variable and each row represents an observation. Standardizing the data format before data analysis enabled clear presentation and efficient reuse of computer code.

**Table 1:** List of 27 papers whose datasets were used for network construction by iDMET

	PMID	Journal	Metabolite detection method	Total number of metabolites incorporated into iDMET (valid metabolite rate)	Ref
1	20861191	Cancer Res	NMR	15 (78.9%)	[21]
2	21853158	PLoS One	GC–MS, LC–MS/MS	213 (85.2%)	[22]
3	21912692	PLoS One	LC–MS	51 (53.15%)	[23]
4	22380946	Cancer Sci	GC–MS	69 ~ 131 (92%–100%)	[24]
5	22421146	Cell Cycle	GC–MS, LC–MS/MS	245 (84.2%)	[25]
6	22628425	Cancer Res	GC–MS, LC–MS/MS	205 ~ 243 (83.3%–91.0%)	[26]
7	24153255	BMC Syst Biol	GC–MS	40 ~ 42 (95.0%–100%)	[27]
8	24952473	Oncotarget	LC–MS/MS	46 (74.2%)	[28]
9	25880539	J Ovarian Res	GC–MS, LC–MS/MS	149 (83.2%)	[29]
10	26023239	J Biol Chem	CE-TOFMS	112 (96.6%)	[30]
11	26311851	J Biol Chem	GC–MS, LC–MS/MS	287 (83.9%)	[31]
12	26318292	Oncotarget	CE-TOFMS	63 (87.5%)	[32]
13	26415588	Mol Cell Endocrinol	GC–MS	70 (86.4%)	[33]
14	26508589	Sci Rep	GC–MS	30 ~ 31 (88.2%–93.9%)	[34]
15	26623558	Oncotarget	GC–MS, LC–MS/MS	117 (87.3%)	[35]
16	26637368	Mol Cancer Ther	LC–MS	193 (85.4%)	[36]
17	26766592	Cancer Cell	GC–MS, LC–MS/MS	491 (85.1%)	[37]
18	26886430	PLoS One	GC–MS, LC–MS	79 (94.0%)	[38]
19	26980435	J Exp Clin Cancer Res	CE-TOFMS	55–60 (67.9%–69.7%)	[39]
20	27533043	Mol Carcinog	1H-NMR	9 (24.3%)	[40]
21	29084919	Clin Cancer Res	CE-TOFMS	70 (73.7%)	[41]
22	30026261	Biosci Rep	1H-NMR	143 (40.5%)	[42]
23	30482722	EBioMedicine	1H-NMR	51 (85.4%)	[43]
24	30538212	Aging (Albany, NY)	GC–MS, LC–MS/MS	227 (61.9%)	[44]
25	30830323	Metabolomics	GC–MS	52 (51.0%)	[45]
26	30903027	Nat Commun	LC–MS	30 (71.4%)	[46]
27	31068703	Nat Med	LC–MS	89 (39.5%)	[47]

Detection method: gas chromatography (GC), liquid chromatography (LC), mass spectrometry (MS), tandem mass spectrometric (MS/MS), time-of-flight mass spectrometry (TOFMS), nuclear magnetic resonance (NMR), capillary electrophoresis (CE). Search date: February 2020. Numbers (%) in parentheses represent rates of metabolites remaining after matching all metabolites that appear in each paper with the list of metabolite names and synonyms that we prepared (i.e., “valid metabolites” for this study)

### Overview of iDMET

**Fig. 2:**
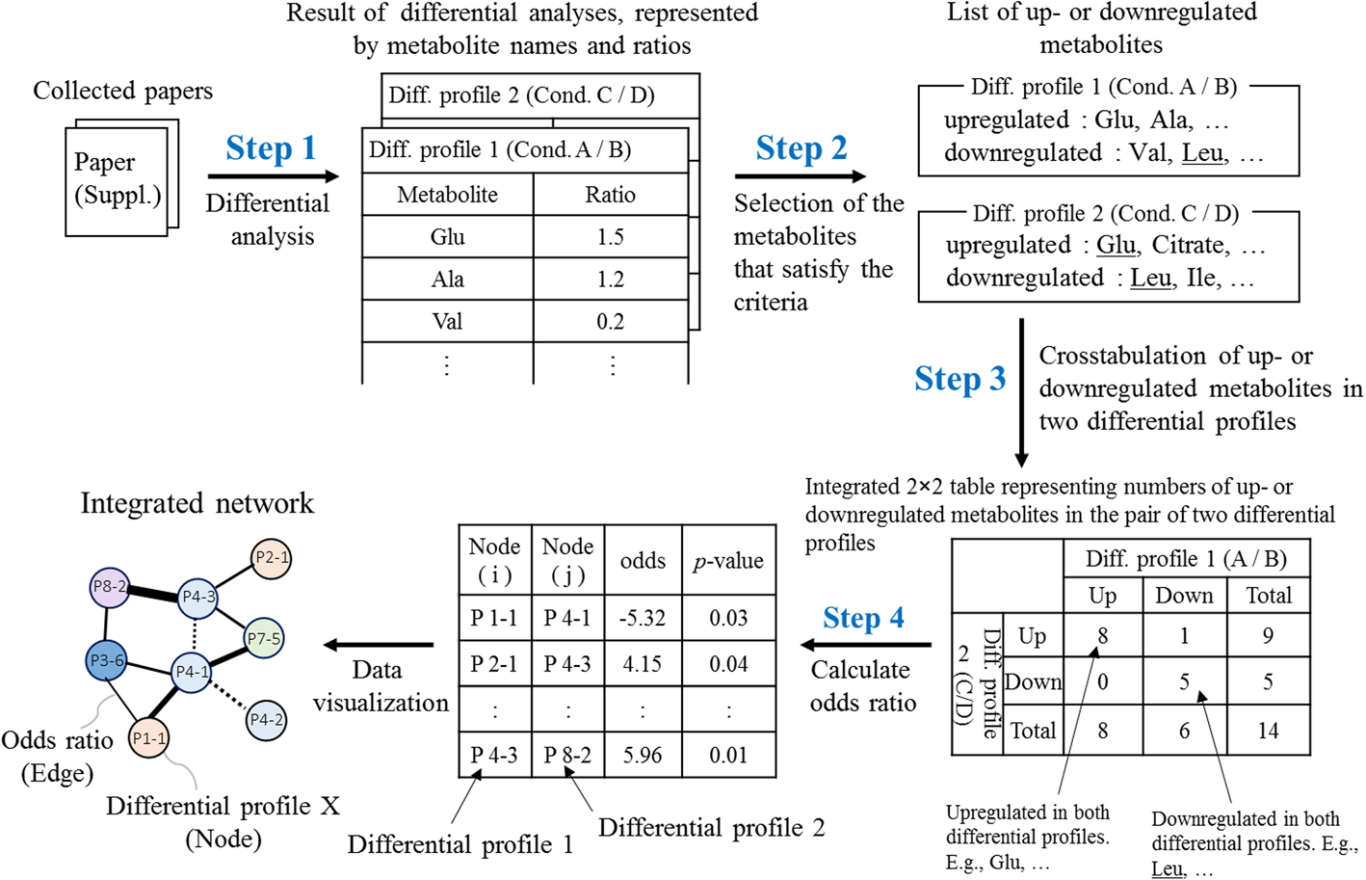
Overview of iDMET for construction of differential metabolomic network based on the published metabolomic datasets. The procedure in iDMET consists of four main steps. The metabolomic data collected from papers or repositories are subjected to differential metabolomic analyses (step 1) to calculate the ratios of metabolite levels among the possible pairs of all conditions that appear in each paper. Step 1 can be omitted if the ratios are provided in the papers. Based on the specified thresholds of the ratios, up- and downregulated metabolites for each comparison of condition pair (designated as “differential metabolomic profile”) are selected (step 2). Then, for all possible pairs of comparisons, a 2 × 2 contingency table counting the number of metabolites that are upregulated (or downregulated) based on one differential analysis while upregulated (or downregulated) in the other differential analysis is generated. For each table, the odds ratio and *p*-value are calculated (step 3). Pairs of differential metabolomic profiles having remarkable odds ratios and *p*-values are selected and visualized as a network where each node represents a comparison (step 4). An edge between a pair of differential metabolomic profiles denotes that the upregulated (or downregulated) metabolites in one differential analysis significantly overlap with those that are upregulated (or downregulated) in the other differential analysis. Further details on the network visualization are given in the legend of Fig. 4

We developed iDMET, a network-based approach for integrating multiple sets of metabolomic data. The overall procedure of iDMET is shown in Fig. 2. iDMET has the advantage of being able to integrate and compare data obtained at different facilities and from different samples even if the absolute metabolite levels (i.e., data matrix of metabolomic profile) are not available. It only requires relative changes of metabolite levels (“differential metabolomic profile”). Steps 1 and 2 are the process of organizing data. We collected supplementary data from papers or repositories to generate a list of variable metabolite names and their values. Steps 3 and 4 are computational processes for network generation. We calculated the similarity of each pair of differential metabolomic profiles based on the information generated in step 2 and visualized the relationships among differential metabolomic profiles as a network (Fig. 2).

Before incorporating metabolomic profiles into iDMET, we needed to convert identifiers of analytes reported in each study to common metabolite names. Thus, we manually created an initial list of the common metabolite names and their synonyms. If a metabolite name that appears in each study was not on our initial list, we expanded the list by manually adding the metabolite name and as many of its synonyms as possible using PubChem. We manually corrected the character if any incorrect characters (extra spaces, garbled characters, and misspelling of metabolite names, etc.) were included in the data. We did not use analytes with only m/z or retention time (but not metabolite name) given. Details of each step are described below.

### Data curation (step 1)

The data were collected mainly from tables and supplementary files of the 25 articles, and two sets of data were collected from the repository. These data included various types of data, such as matrices of samples and metabolites, and tables featuring metabolite names with the ratios of changes in their levels between two groups, and *p*-values. To analyze these data using iDMET, we manually converted all data into tables consisting of the names of metabolites, and the corresponding ratios of the differences in their levels between two groups. If there were three groups or more, the ratios were calculated for all combinations and used to generate the network.

### Data integration (step 2)

From the tables created in step 1, we selected metabolites whose ratios were higher than the upper threshold (“upregulated metabolites”) or lower than the lower threshold (“downregulated metabolites”). In this study, we set the following thresholds: ratio > 1.2 (upper threshold) or ratio < 1/1.2 (lower threshold), and alternatively as a more stringent option, ratio > 1.5 or ratio < 1/1.5. In this study, we mainly focus on the former thresholds.

### Similarity assessment of two differential metabolomic profiles (step 3)

A 2 × 2 crosstabulation table was created using the number of metabolites that were up- or downregulated in a pair of differential metabolomic profiles. The odds ratio calculated based on the four numbers in the table [odds ratio = (*m*_1,1_/*m*_1,2_)/(*m*_2,1_/*m*_2,2_) = (*m*_1,1_·*m*_2,2_)/(*m*_1,2_·*m*_2,1_), where *m*_*i,j*_ represents the number in the table] was used as the degree of correlation between the pair. It is analogous to enrichment score, which is frequently used in functional and pathway enrichment analyses. Odds ratio may work efficiently to capture a significantly correlated pair if an up- or downregulated level of metabolites beyond the threshold is not important. If any of the four numbers was 0, 0.5 was added to all four numbers, so that the odds ratio could be calculated. We performed this calculation for all pairwise combinations of differential metabolomic profiles. For interesting pairs of differential metabolomic profiles, we also checked the correlation coefficient of their differential profiles, besides the odds ratio. Finally, we created a graph adjacency matrix, in which each element contains the value of the odds ratio for each pair of differential metabolomic profiles.

### Visualizing network (step 4)

The weighted network was visualized based on the graph adjacency matrix. Each node in the network represents the pair of metabolomic profiles from the same publication, where the numbers of up- and downregulated metabolites were calculated, which represents a differential metabolomic profile. Each edge represents the similarity of the pair of differential metabolomic profiles corresponding to the connected node pair. The thickness of the edge represents the odds ratio. However, when the result of the chi-squared test for the edge was not significant (*p*-value > 0.05), the edge was removed. Unconnected nodes were removed and the network was visualized in Cytoscape version 3.7.2 [48, 49].

### Statistical analysis

For simple merging and analyses of metabolomic data, we chose the widely used tool MetaboAnalyst 4.0 [50]. There were large numbers of missing values, as is often the case in a typical metabolomic dataset, and they were replaced by one-fifth of the minimum positive value among the corresponding metabolite levels. For normalization of the dataset, the following settings were used: sample normalization, quantile normalization; data scaling, auto scaling. Hierarchical clustering with a heat map and dendrograms were used to investigate patterns of metabolomic profiles in the dataset. The “gplots” package [51] in R software version 3.3.3 was used to visualize missing values in the datasets. Other statistical analyses were performed using R software and the “igraph” package [52] was used to conduct network analyses before visualizing the network using Cytoscape [48, 49].

## Results and discussion

### Characteristics of the included studies

Our PubMed and repository search conducted in February 2020 found 330 relevant studies, with 324 found by PubMed search and 6 found in MetabolomeXchange. Most papers were available under an open access scheme (Fig. 1). A total of 298 articles were review or methodology papers or those without quantitative values provided. Five articles did not contain useful metabolomic data as they focused on a single biomarker or lipidomics. The final number of articles valid for this study was thus 27. The 27 datasets curated for this study are summarized in Table 1. The formats of these publicly available datasets were very different. For example, we found that only 5 out of the 27 articles included data in a csv file or in a text document that could be easily imported for computations. In the other studies, the results were either embedded in the main text or provided as a PDF supplement.

There is an issue of variety in how the data provided in each article were processed to control their quality. We found that only 6 out of 27 articles excluded metabolites based on technical variability such as coefficient of variation and relative standard deviation within QC samples, while some articles gave little information about the metabolite exclusion criteria. Handling missing values is also one of the major procedures for pre-processing. The most common approach to treat a missing value of a metabolite was to replace these by the smallest value among the levels of the corresponding metabolite. Only 8 out of 27 articles reported imputing the missing values (Additional file 2: Table S2). The Metabolomics Standards Initiative (MSI) proposed minimum criteria (e.g., metadata, sample preparation, data processing) for reporting metabolomic analysis in order to facilitate data sharing [53]. However, it has been reported that the data pre-processing and how the results were reported did not follow any standardized procedure [54, 55]. Playdon et al. reported that, if the measurement was done by contractors, the data pre-processing methods applied were not always available to the user [54].

**Table 2:** List of significant node pairs that constitute the network

Pair		Platform		Cancer type		Ratio > 1.2, ratio < 1/1.2	
Node 1	Node 2	Node 1	Node 2	Node 1	Node 2	Odds	*p*-value
P17-1	P24-1	GC–MS, LC–MS/MS	GC–MS, LC–MS/MS	Kidney	Kidney	8.95	4.2E − 32
P7-1	P14-7	GC–MS	GC–MS	Colon	Colon	6.71	0.0018
P7-1	14–14	GC–MS	GC–MS	Colon	Colon	6.71	0.0018
P7-1	P14-1	GC–MS	GC–MS	Colon	Colon	6.57	0.0028
P7-1	P14-13	GC–MS	GC–MS	Colon	Colon	6.57	0.0028
P7-1	P14-10	GC–MS	GC–MS	Colon	Colon	6.51	0.0033
P7-2	P14-2	GC–MS	GC–MS	Colon	Colon	6.29	0.0019
P7-1	P14-5	GC–MS	GC–MS	Colon	Colon	6.20	0.0075
P7-2	P14-10	GC–MS	GC–MS	Colon	Colon	6.04	0.0040
P7-2	P14-5	GC–MS	GC–MS	Colon	Colon	5.91	0.0059
P7-1	P14-2	GC–MS	GC–MS	Colon	Colon	5.75	0.0088
P7-1	P14-4	GC–MS	GC–MS	Colon	Colon	5.67	0.0094
P10-2	P18-3	CE-TOFMS	GC–MS, LC–MS	Lung	Prostate	4.81	0.0040
P7-2	P14-1	GC–MS	GC–MS	Colon	Colon	4.58	0.0294
P7-2	P14-13	GC–MS	GC–MS	Colon	Colon	4.58	0.0294
P7-2	P14-14	GC–MS	GC–MS	Colon	Colon	4.46	0.0194
P7-2	P14-7	GC–MS	GC–MS	Colon	Colon	4.17	0.0375
P6-2	P25-1	GC–MS, LC–MS/MS	GC–MS	Kidney	Breast	4.17	0.0492
P16-1	P17-1	LC–MS	GC–MS, LC–MS/MS	Colon	Kidney	− 4.68	0.0000
P17-1	P18-3	GC–MS, LC–MS/MS	GC–MS, LC–MS	Kidney	Prostate	− 4.46	0.0021
P6-2	P18-3	GC–MS, LC–MS/MS	GC–MS, LC–MS	Kidney	Prostate	− 4.29	0.0022
P5-2	P11-7	GC–MS, LC–MS/MS	GC–MS, LC–MS/MS	Lung	Leukemia	− 4.15	0.0394
P16-2	P17-1	LC–MS	GC–MS, LC–MS/MS	Colon	Kidney	− 4.08	0.0002

Based on the specified thresholds of the ratios, differential metabolomic profile pairs having remarkable odds ratios and *p*-values were selected (*p* < 0.05, odds > 4). The odds ratio shows a high positive (or negative) value if the metabolites were consistently upregulated (or downregulated) in the differential metabolomic profile pair (for details, see Fig. 2, Additional file 1: Table S1, and methods)

Each article used different metabolite names and IDs for the same metabolite because metabolites have many synonyms but lack a standardized nomenclature. Therefore, the metabolite names that appear in each article were converted to standard names in the list (for further details, see methods). Over 70% of the metabolites provided in each study successfully matched with one of the metabolites in our prepared list with synonyms (Table 1). In other words, 30% of the metabolites failed to be converted to any of the metabolites in our list. Metabolites with ambiguous annotations (i.e., unknown metabolites or an inability to discriminate between the isomers) were not used.

The analytical process was less homogeneous; the authors used various techniques, including nuclear magnetic resonance imaging (NMR) spectroscopy, LC, GC, and CE (for full details, see Table 1). The analyzed samples include plasma, urine, tissue, culture cells, and culture medium. There are many factors related to sample handling that may influence measured metabolite levels, including the medium used for cell culture, storage period in the freezer, centrifugation conditions, and temperature of storage prior to metabolite extraction, although most samples were frozen at − 70 °C to − 80 °C until they were extracted and analyzed.

### Simple merging of data matrices was not an efficient strategy for multiple metabolomic data integration

**Fig. 3:**
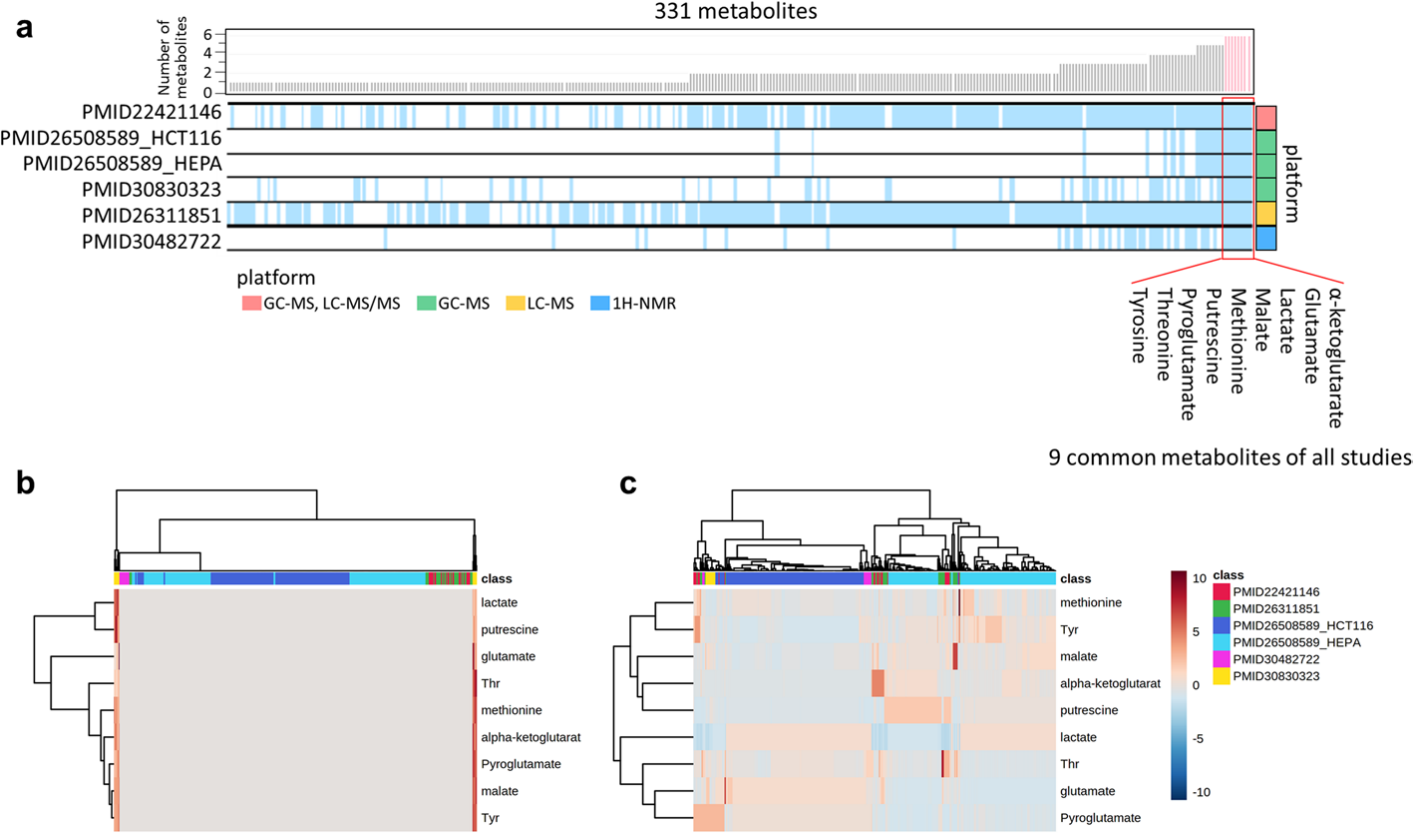
Inefficiency of simply merging multiple metabolomic profiles. Six data matrices of metabolomic profiles from five publications were merged and visualized. **a** An overview of the investigated metabolites that overlap across the six datasets. The rows correspond to each metabolomic dataset and the columns correspond to metabolites. Blue vertical line indicates that the corresponding metabolite is a target of the analysis in at least one sample in the dataset. White spaces represent metabolites that are not reported in the given studies or where a failure to detect their peak occurred due to a low metabolite concentration. The bar plots above represent the numbers of datasets that include the given metabolite, which were used to sort the metabolites in this heat map; nine metabolites that are covered by all six datasets are indicated on the right. Colored squares on the right represent the platform types (see also legend). **b**, **c** Hierarchical clustering of the levels of nine common metabolites was performed based on Pearson’s correlation coefficient as a distance metric and average linkage as a clustering algorithm. Each column represents the metabolomic profile from the literature specified by a color on the top, and each row represents each metabolite in the profile. The color of each cell represents the metabolite level shown by a color gradient bar on the right (low: blue, high: red). Auto scaling was applied for data pre-processing. **b** indicates that metabolomic profiles from PMID30830323 (columns marked with yellow at both ends) likely show extreme values compared with the other profiles. For (**c**), quantile normalization was applied before auto scaling. Then, it is clear that metabolomic profiles were probably clustered by the literature from which the profiles were derived. (a) was generated by R software and the “gplots” package [51]. **b**, **c** were generated using MetaboAnalyst [50]

Treating missing values is an important task during metabolomic data analysis. In most cases, data are missing because of an actual absence of the compound in the samples, a failure to detect peaks of low-concentration metabolites, or the metabolite corresponding to the missing value not being one of the targets for analysis by the instruments in the study. The number of metabolites included in each study ranged from 15 to 491 (Table 1). Notably, some of the 27 studies did not fully report their collected metabolomic data. Only 5 (18.5%) of the 27 studies reported data on all measured metabolites as real-value matrices (Figs. 1, 3a). This is in contrast to the proteomic and transcriptomic data, where full dataset deposition in repositories is often required.

These six sets of data matrix of metabolomic profile from five studies were used to investigate the efficiency of simple merging for metabolomic analyses. The heat map in Fig. 3a represents how the investigated metabolites overlap across the six datasets. If the level of specific metabolite was reported in the specific study, the corresponding cell of the heat map was colored blue. Otherwise, the position was left empty, which implies that the metabolite level was not reported or there was a failure of peak detection due to a low metabolite concentration. From the abundant white “empty” spaces, it is clear that the reporting of metabolites was far from complete. We noted that the levels of only nine metabolites were reported in at least one sample in each of the five studies. This suggested that, for simply merging metabolomic data, we could perform classical statistical analyses such as hierarchical clustering only for a small proportion of metabolites (only nine metabolites) because there were few common metabolites when we simply merged metabolomic data derived from the different metabolomic platforms. Also note that the clustering result may be highly dependent on the small number of specific metabolites selected.

Furthermore, to explore how the nine common metabolites (in five studies) are altered across cancer types, we used hierarchical clustering visualized by heat maps and dendrograms for simply merged metabolomic data with the imputation of missing values (Fig. 3b, c). Prior to analyses, metabolomic data should be normalized to exclude technical variations originating from various factors including sample pre-processing and measurement by instruments, especially when integrating results from different laboratories [56]. Data scaling is used to adjust biases among various metabolomic data. Also in our work, data were subjected to auto scaling (Fig. 3b) before further analysis. Metabolomic profiles shown by two columns at both ends (denoted by yellow in the class bar) in Fig. 3b are from publication PMID30830323 [45] and appeared to show extreme values compared with the other metabolomic profiles in the same figure, and such values visually obscured other metabolomic profiles. These results suggest that, if only auto scaling were applied, inter-study bias would be prominent and hide any other characteristic patterns of metabolite levels in this integrated metabolome dataset. Therefore, quantile normalization was applied before auto scaling to mitigate the extreme values (Fig. 3c). As a result, the metabolomic profiles were grouped primarily by study, while samples originating from different studies were rather dispersed (Fig. 3c). Ideally, metabolomic profiles should be clustered based on cancer type, but the clustering of metabolomic profiles from the same cancer type was much less evident. We noticed that two metabolomic profiles, PMID30482722 [43] and PMID30830323 [45], were from the same cancer type (breast cancer), but they were not grouped into a single cluster, although the separation may be due to differences in the cancer subtypes.

These results suggest that simple merging of metabolomic data with quantile normalization that corrects biases in overall metabolite level distributions among different studies is still inefficient for integrating multiple sets of metabolomic data derived from different metabolomic platforms.

### Pairwise integration and network generation using common differential metabolites

**Fig. 4:**
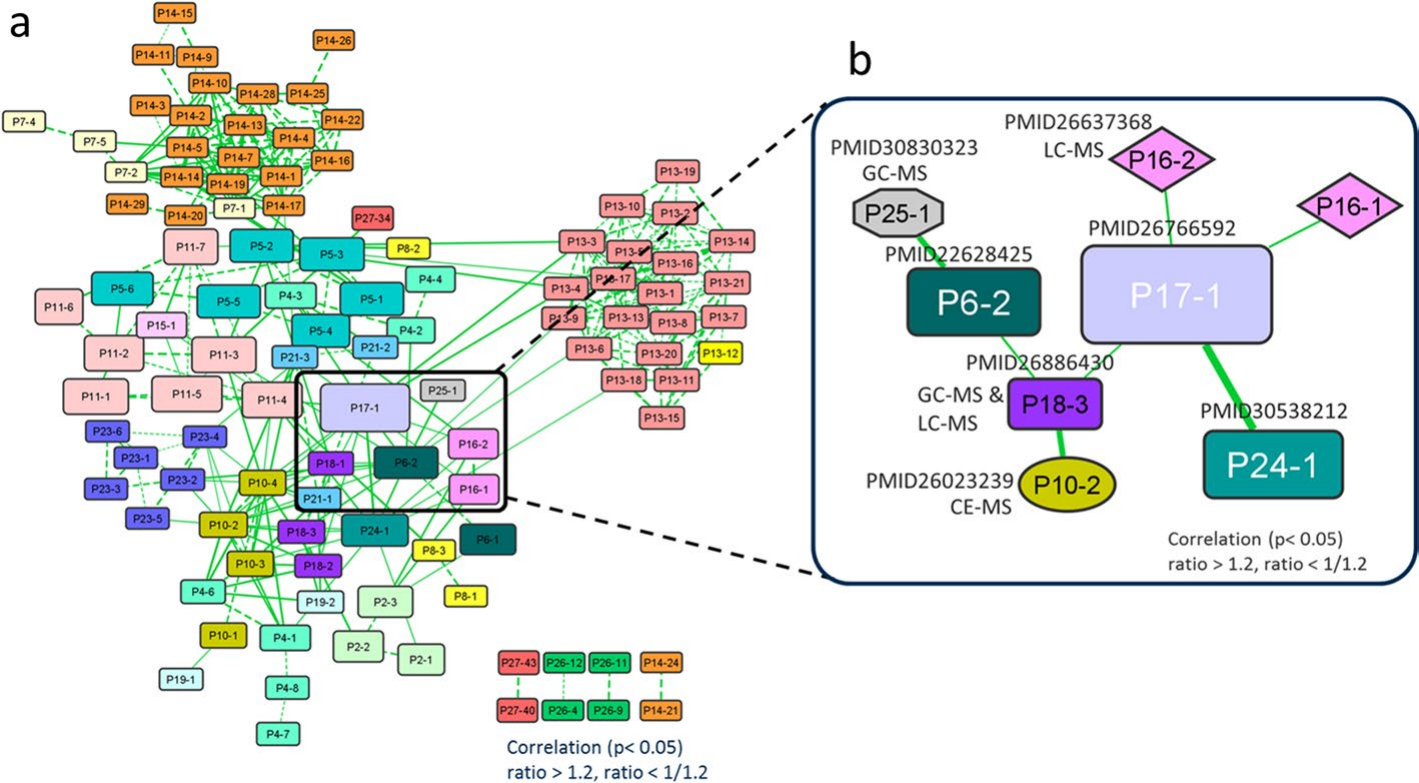
Differential metabolomic network based on the published cancer metabolomic datasets. Metabolomic data from the list of papers in Table 1 were used to generate the network in (**A**). Each node represents a comparison of a metabolomic profile pair (differential metabolomic profile). A pair of nodes are connected by an edge if the corresponding pair of differential metabolomic profiles are correlated (see also Fig. 2). The node color reflects the source article, where the same color denotes that the corresponding source data are from the same paper. The widths of edges indicate odds ratio weights. The node size depicts the total number of differential metabolites for each dataset. The network connects metabolomic data not only from the same laboratory (denoted by dotted edges), but also those from different laboratories (denoted by solid edges). The subnetwork of (**a**) that contains a strong connection between P17-1 and P24-1 is magnified in (**b**) and further assessed in Fig. 5. The network was visualized by Cytoscape

As described in the previous section, we were only able to integrate a small number of metabolites in a limited number of studies (Fig. 3). In this section, we introduce iDMET to integrate all of the data in a pairwise manner, calculating all possible pairwise combinations of pairs of differential metabolomic profiles where each differential profile represents changes in the metabolite levels calculated based on the comparison between two conditions (Fig. 2). This allows us to use a larger number of metabolites showing correlated changes in two differential metabolomic profiles to determine the relationships among the compared pairs (Fig. 4a). The iDMET method has several advantages compared with simple merging of data. For example, it requires only ratios of metabolite levels between each condition pair as input data, so data matrices of metabolomic profiles are unnecessary. In addition, iDMET can further build large-scale networks enabling the exploration of multiple conditions simultaneously.

Here, we applied iDMET to various metabolomic data from 27 articles on cancer metabolomics. At the threshold of ratio > 1.2 (or ratio < 1/1.2) and *p* < 0.05 in the chi-squared test (see Fig. 2 and methods), we obtained 348 pairs of differential metabolomic profiles. Among them, there were 236 and 112 pairs of differential metabolomic profiles where the two datasets associated with each pair were from the same and different papers, respectively (Fig. 4a). At the more stringent threshold of ratio > 1.5 (or ratio < 1/1.5) with the same *p*, we obtained 192 pairs, of which 35 pairs had datasets from different papers. Thus, pairs of about 70%–80% of the obtained dataset pairs were from the same paper. This was due to the fact that the same set of metabolites is usually analyzed in multiple conditions in a single paper, and the number of metabolites showing correlated changes among different conditions in the same paper is apparently larger than that among different conditions over different papers, since the number of targeted metabolites common to two different papers is usually low, as we discussed in the previous subsection. To avoid this bias, we decided to focus only on pairs from different papers in this study. The top 20 pairs sorted based on odds ratios are shown in Table 2 and Additional file 1: Table S1. We note that we used raw *p*-values for the chi-squared test, although, in the future, we are planning to adjust *p*-values so that the results will be statistically more robust.

### The network successfully identified biologically relevant pairs of differential metabolomic profiles

**Fig. 5:**
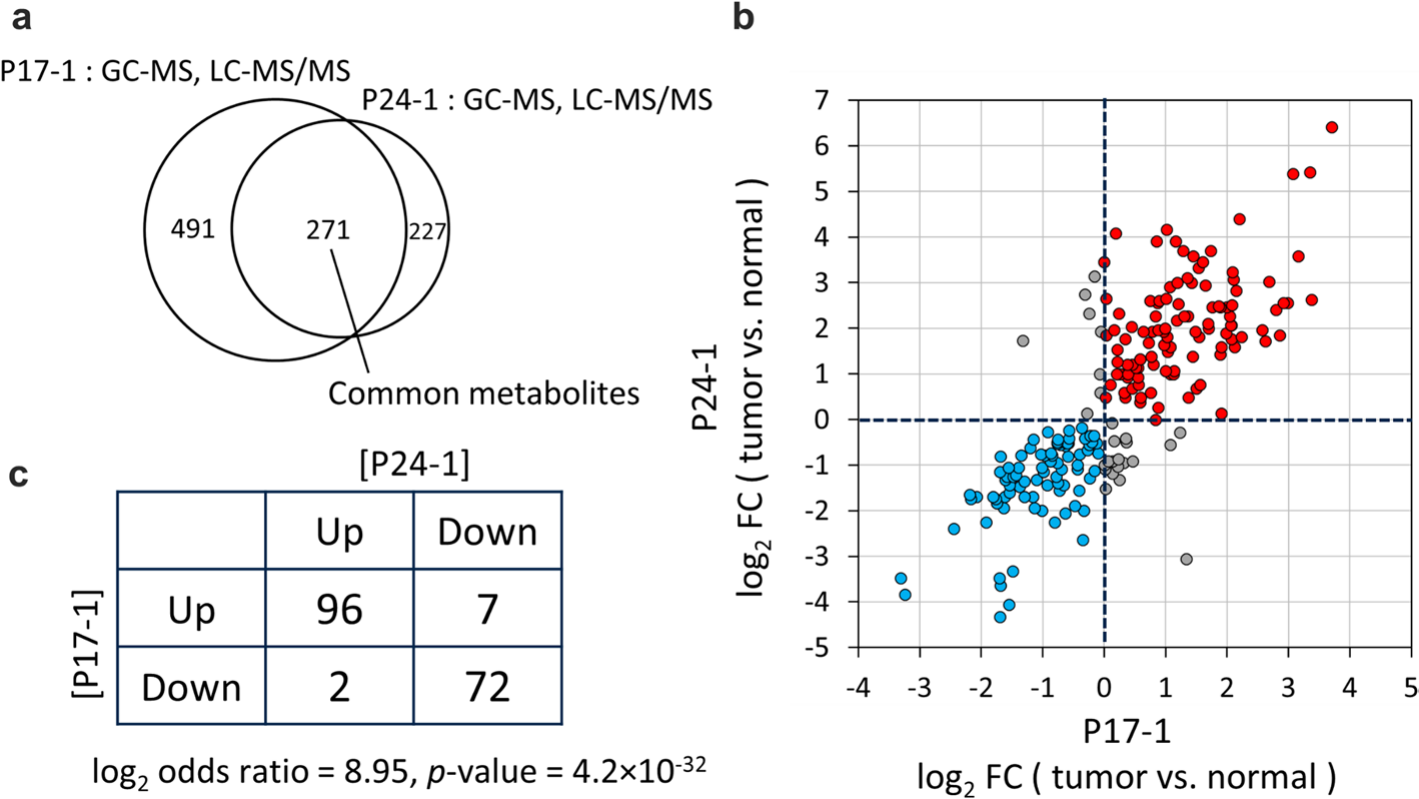
Assessment of connection between P17-1 and P24-1 in the network. **a** Venn diagram showing the number of metabolites common to both of the two cohort studies on which P17-1 [37] and P24-1 [44] were based. In both studies, metabolomic analyses were conducted on human clear cell renal cell carcinoma and normal kidney tissue using GC–MS and LC–MS. **b** Comparison of changes in the level of metabolites in node P17-1 versus node P24-1 (*r* = 0.808, *p* < 0.001, Spearman’s rank test). In both nodes, the changes from normal tissue to tumor were calculated. Red plots represent metabolites found to be upregulated in the tumor group, whereas blue plots represent those found to be downregulated. **c** A 2 × 2 contingency table of the numbers of upregulated and downregulated metabolites in the two nodes (log2 odds ratio = 8.95, *p*-value = 4.2 × 10^−32^). The table was created based on (**b**). See Fig. 2 for details on the table creation

The top pair in Table 2 was the edge between nodes 17–1 (P17-1) [37] and 24–1 (P24-1) [44]. (The node ID is in the format Px-y, where x and y represent publication and pair of datasets within the study, that is, a differential metabolomic profile, respectively.) Its log2 odds ratio was 8.95 (*p*-value = 4.2 × 10^−32^). Both of their corresponding original publications describe cohort studies that compared metabolomic profiles of clear cell renal cell carcinoma (ccRCC) and normal kidney tissues (Fig. 4b, 5, Table 2, Additional file 1: Table S1). Thus, the biological conditions in which metabolomic profiles were obtained are highly relevant in these two studies, which justifies the inclusion of this edge in our network. Among the collected publications (Table 1), the above-mentioned two studies were the only pair obtained for the same disease using the same sample type. It is thus reasonable that this pair is the most significant in Table 2 and Additional file 1: Table S1. Overall, 217 metabolites were common to both of the two studies (Fig. 5a) and the changes of their levels among two differential profiles, namely, tumor vs. normal tissue in each study, were used to assess the significance of the pair of differential profiles. There was a positive correlation between fold change of metabolite levels in P17-1 and P24-1 (*r* = 0.808, *p* < 0.001, Spearman’s rank test; Fig. 5b). Figure 5c shows a 2 × 2 contingency table created based on the data in Fig. 5b. It was used to assess the statistical significance of the similarity of differential metabolomic profile pairs and to determine whether each profile pair should be connected by an edge, according to the thresholds of log2 odds ratio and *p*-value. There were 96 and 72 metabolites whose levels were up- and downregulated, respectively, in tumor compared with the levels in normal tissue in both studies. The number of metabolites showing correlated changes (96 + 72 = 168) was far greater than that showing uncorrelated changes (2 + 7 = 9, Fig. 5c).

The reason for the high log2 odds ratio resulting from a high correlation may be that the employed analytical instruments were the same (GC–MS and LC–MS/MS) and the measurements of the two studies were carried out at the same research institute [37, 44]. Consequently, the measured metabolomic profiles were less affected by the analytical conditions, and we were able to see that the change of metabolomic profiles among normal versus tumor samples observed in one study was clearly reproduced in the other study, with similar biological conditions.

The clinical background of the patients (e.g., age, sex, BMI, stage of the disease) in the two cohort studies was heterogeneous and we thought that the metabolite levels may vary depending on the participant [37, 44]. However, we were able to observe a clear correlation (Fig. 5b, c). Although the absolute amounts of the metabolites may vary from patient to patient, the direction of the regulation in tumor compared with normal tissue (i.e., either up- or downregulated) appeared to be fairly consistent throughout the patients.

The next significant pair was nodes 7–1 (P7-1) [27] and 14–7 (P14-7) [34]. In addition, many other pairs from these two papers were also identified (Fig. 4a, Table 2, Additional file 1: Table S1, 2nd to 12th and 14th to 17th node pairs). We found that the above two papers were published from the same laboratory, and some of the data were shared between the two publications used in the analysis, so it is reasonable that this pair ranked high in Table 2.

### Discovering novel connection of biological phenomena from the network

**Fig. 6:**
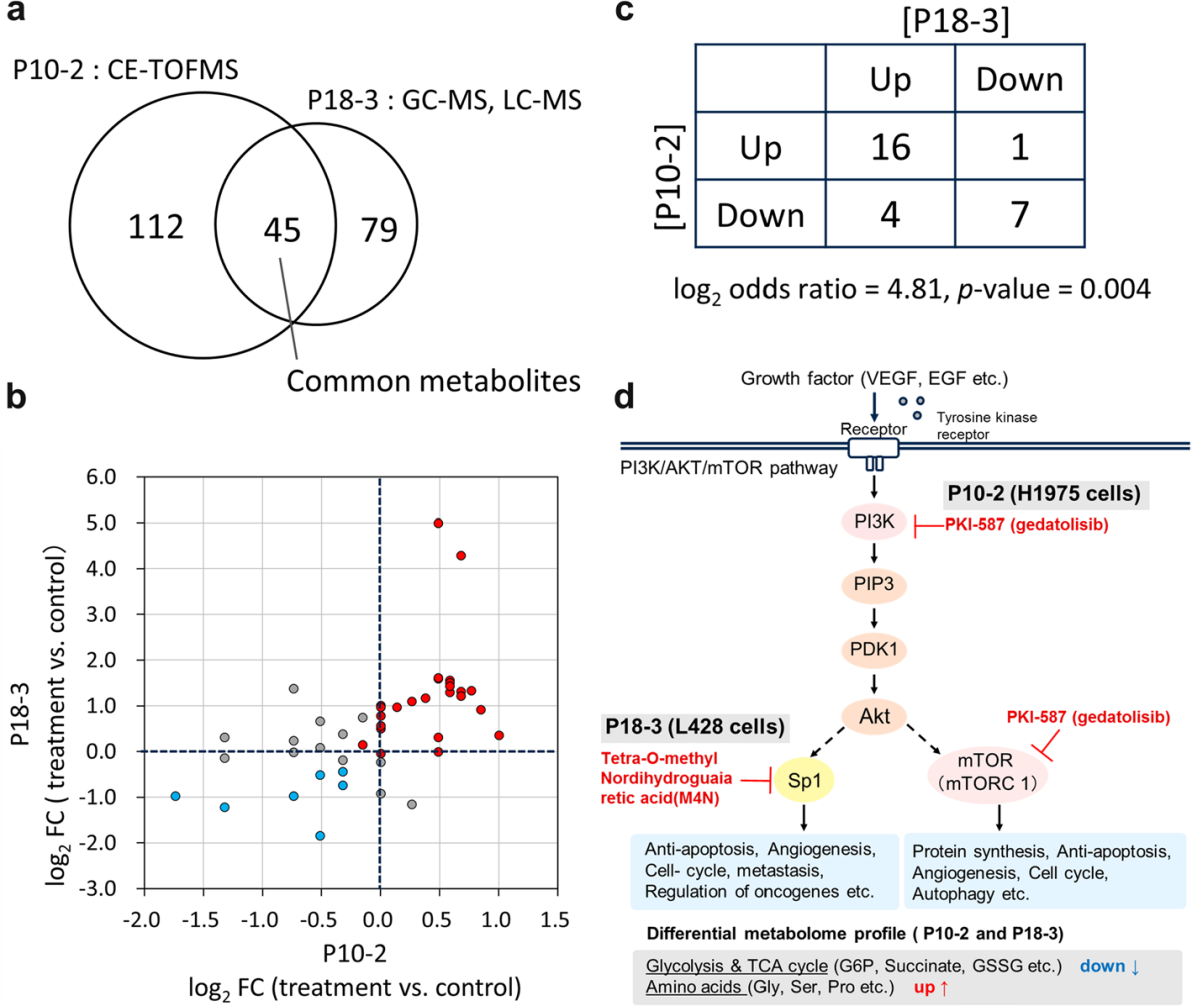
Discovery of novel connections between two studies. **a** Venn diagram showing the number of metabolites common to both of the two studies on which P10-2[30] and P18-3 [38] were based. In both studies, metabolomic analyses were conducted using CE-TOFMS, GC–MS, and LC–MS. **b** Comparison of changes in the level of metabolites in P10-2 versus P18-3 (*r* = 0.656, *p* < 0.001, Spearman’s rank test). P10-2 represents the differential metabolomic profile in H1975 cells (H1975; human lung adenocarcinoma cell line) after treatment with PKI-587 (gedatolisib). P18-3 represents the metabolomic profile in L428 cells (L428; human Hodgkin’s lymphoma cell line) after treatment with tetra-O-methyl nordihydroguaiaretic acid (M4N). Their controls were the cells before drug treatment. In both nodes, the changes from control to drug-treated were calculated. Red plots represent metabolites found to be upregulated in the tumor group (treatment group), whereas blue plots represent those found to be downregulated. **c** A 2 × 2 contingency table of the numbers of upregulated and downregulated metabolites in the two nodes (log2 odds ratio = 4.81, *p*-value = 0.004). The table was created based on (**b**). See Fig. 2 for details on the table creation. **d** Schematic representation of PI3K/AKT/mTOR pathways and the point of inhibition by the drugs used in the two studies. Metabolite classifications to which simultaneously upregulated or downregulated metabolites belong are shown at the bottom. PI3K, phosphatidylinositol 3-kinase; PIP3, phosphatidylinositol 3-phosphate; PDK-1, 3-phosphoinositide-dependent protein kinase 1; Akt, protein kinase B (PKB, also called Akt); mTOR, mammalian target of rapamycin; Sp1, Specificity protein 1

We further explored edges in the network associated with strong correlations of a pair of differential metabolomic profiles, each of which represents an upregulation or downregulation in cancer relative to controls (change after drug treatment). We also explored how consistently metabolites were altered across drug types. There was a strong positive correlation between nodes 10–2 (P10-2) and 18–3 (P18-3) (Fig. 4b, 6, Table 2, and Additional file 1: Table S1). The original two studies corresponding to these two nodes investigated the effects of drug treatments (two drugs for P10 and one drug for P18) on metabolomic profiles using various human cancer cell lines, including those of lung adenocarcinoma, prostate carcinoma, and Hodgkin’s lymphoma [30, 38]. Both studies conducted metabolomic analyses using CE-TOFMS, GC–MS, and LC–MS/MS (Fig. 6a). Detailed descriptions of the experimental methods, cell lines, and used drugs can be found in Additional file 3: Table S3 as well as in the original articles.

P10-2 represents the differential metabolomic profile in H1975 cells (H1975; human lung adenocarcinoma cell line), which compares metabolite levels between before and after treatment with PKI-587 (gedatolisib) [30]. P18-3 represents the differential metabolomic profile in L428 cells (L428; human Hodgkin’s lymphoma cell line), which compares metabolite levels between before and after treatment with tetra-O-methyl nordihydroguaiaretic acid (M4N) [38]. The controls for these differential analyses were set as the metabolomic profiles before the drug treatments (Fig. 6b). There were 45 metabolites common to both of the two studies (Fig. 6a) and the changes of metabolite levels upon drug treatment were highly correlated (*r* = 0.656, *p* < 0.001, Spearman’s rank test; Fig. 6b). The number of up- and downregulated metabolites in both studies (16 + 7 = 23) was greater than that showing inconsistent changes between the two studies (1 + 4 = 5, Fig. 6c). The metabolites that showed upregulation upon treatment in both studies included tyrosine, tryptophan, glycine, proline, and phenylalanine. Those that showed downregulation in both studies included glucose-6-phosphate, glucose-1-phosphate, succinate, and GSSG.

We noticed that both drugs (M4N and gedatolisib) inhibit factors in the PI3K/AKT/mTOR pathway (Fig. 6d), which is critical for the regulation of aerobic glycolysis and cell proliferation [30]. This pathway is abnormally activated in cancer cells and it has marked effects on tumor cell maintenance and survival, protein synthesis, and altered metabolomic pathways. The drug M4N suppresses Specificity protein 1 (Sp1), which is a transcription factor that plays a role in the regulation of oncogenes required for tumor survival and progression [57, 58]. Gedatolisib is a dual inhibitor of phosphatidyl inositol 3-kinase (PI3K) and mammalian target of rapamycin (mTOR) [30, 59, 60]. The group of downregulated metabolites in both studies are products of glycolysis and the TCA cycle and the group of upregulated ones are amino acids (Fig. 6c, d). Both of the drug targets, Sp1 and mTOR, regulate pathways such as the cell cycle and apoptosis (Fig. 6d bottom), which have major impacts on overall cellular processes. We speculate that glycolysis and the TCA cycle were particularly affected by such treatment. The upregulation of amino acids can be explained by the inhibition of Sp1 and mTOR, both of which have roles in suppressing autophagy, which may upregulate amino acid levels by autophagy. This connection between P10-2 and P18-3 may be justified by its aforementioned biological relevance, that is, two drugs affect the same pathway, which can be a target for further experimental investigation to determine the similarity of molecular reactions initiated by gedatolisib and M4N.

Thus, iDMET discovered a connection between different cancer cells, each of which was treated with different drugs. This example of discovering a novel connection implies that, by adding a much larger number of differential metabolomic profiles from other publications, we may be able to discover more novel connections (In the current study, the discoveries were made based on only 27 publications).Therefore, we suggest that iDMET, which focuses on the relationships among differential metabolomic profiles, might be a useful tool for discovering novel relationships between biological reactions including drug responses.

### Current issues of publicly available data for iDMET

We note that the nomenclature of metabolites is an important technical issue for our approach, given that the integration of metabolomic profiles from different studies is based on metabolite IDs or names. However, for each metabolite, there are usually synonyms and multiple IDs from different databases. If the matching of metabolite names or IDs from two studies fails, it will result in an undercount of metabolite overlap between the two studies, which often happened in the current study. Therefore, we might have missed important cancer-associated metabolites. Once this problem is resolved through standardization of IDs, metabolite names, and nomenclature, we can perform more accurate network analyses.

It should also be noted that publicly available metabolomic datasets were limited, which is a particular problem in metabolomics [7]. The deposition of matrix data of metabolomic profiles to public repositories is not yet common in metabolomics, partly because it is not always mandated by scientific journals. Generally, public availability and reuse of datasets is important because it is considered to be a good scientific practice (e.g., for reproducing the results or for obtaining new findings from published data). As metabolomic repositories (e.g., Metabolomics Workbench [18] and MetaboLights [17]) are improved and more datasets are uploaded, we anticipate that data sharing in metabolomics will improve. By incorporating these datasets into network analysis, we may have a much higher chance of discovering novel relationships between the registered studies.

### Future directions

We note that, since iDMET is a network-based approach of discovering novel relationships between differential metabolomic profiles from different studies, the use of network-based algorithms may boost the discoveries. For example, general subnetwork extraction tools such as CytoCluster [61] may extract sets of metabolomic profiles having important relationships, although for our current dataset, it mainly extracts subnetworks that are composed of metabolomic profiles from the same studies (Additional file 4: Table S4). There are a number of sophisticated algorithms to analyze biological networks [62] and applying appropriate ones to our dataset, which should expand to a much larger size in future, may efficiency of discovery.

## Conclusion

In this study, we developed iDMET, a network-based approach connecting differential analysis for metabolomic data integration. iDMET has the advantage of enabling the integration and comparison of data obtained at different facilities and from different samples, even if the absolute metabolite levels are not available. By applying iDMET to the analysis of cancer metabolomic datasets, we uncovered new associations between drugs that may have effects on similar metabolic reactions, which may lead to a novel hypothesis on the underlying pathway common to these drug responses. We hope that iDMET will help researchers to visualize and integrate complex metabolomic datasets, and thus promote hypothesis generation and verification.

## Supplementary Information

**Additional file 1: Table S1**. List of significant node pairs that constitute the network. We calculated the similarity of each pair of differential metabolomic profiles based on the information generated in step 2 (Fig. 2) and we selected pairs having remarkable odds ratios and *p*-values (*p* < 0.05, odds > 4). We set the following thresholds: ratio > 1.5 (upper threshold) or ratio < 1/1.5 (lower threshold), or alternatively, ratio > 1.2 or ratio < 1/1.2 (for details see Fig. 2 and methods). Match count values represent the number of metabolites common to the given pair. Changed metabolite values represent the number of metabolites that passed the given threshold. The network generated with the threshold ratio > 1.2 (upper threshold) or ratio < 1/1.2 (lower threshold) was investigated in detail in the current study (see also Table 2).

**Additional file 2: Table S2**. Quality control of metabolomic data conducted in each study. Abbreviations are as follows: Ctr, non-cancerous control thyroid; FA, follicular adenoma; FTC, follicular carcinoma; PTC-CV, classical variant of papillary carcinoma; PTC-FV, follicular variant of papillary carcinoma; MTC, medullary carcinoma; ATC, anaplastic carcinoma. The question mark represents that how missing values were dealt with was not clearly described.

**Additional file 3: Table S3**. Experimental information of the two studies P10 and P18. Cell lines and drugs used in each study. An asterisk represents that metabolomic analysis was conducted after the drug treatment.

**Additional file 4: Table S4**. The 12 sub-networks identified by CytoCluster. The node ID is in the format Px-y, where x and y represent publication and differential metabolomic profiles (pair of datasets within the study), respectively.

## Data Availability

The data presented in this study are publicly available at https://github.com/riramatsuta/iDMET.

## Abbreviations

CE: Capillary electrophoresis
LC: Liquid chromatography
GC: Gas chromatography
TOF: Time-of-flight
ccRCC: Clear cell renal cell carcinoma
